# A Routine Neck Massage, a Devastating Stroke: Imaging Clues to Vertebral Artery Dissection

**DOI:** 10.5334/jbsr.4185

**Published:** 2025-12-22

**Authors:** Jie Liu, Peifei Jia, Qingyu Ji

**Affiliations:** 1 Qingpu Branch of Zhongshan Hospital Affiliated to Fudan University, Qingpu, Shanghai, China; 2 Second Affiliated Hospital of Baotou Medical College, Baotou, inner Mongolia, China

**Keywords:** vertebral artery dissection, stroke, neck massage, magnetic resonance angiography, computed tomography angiography

## Abstract

*Teaching point:* In young adults with neurological symptoms after neck trauma, consider vertebral artery dissection. High‑resolution magnetic resonance angiography is superior to CT angiography for diagnosis if the false lumen is thrombosed.

## Case History

**Figure 1:**
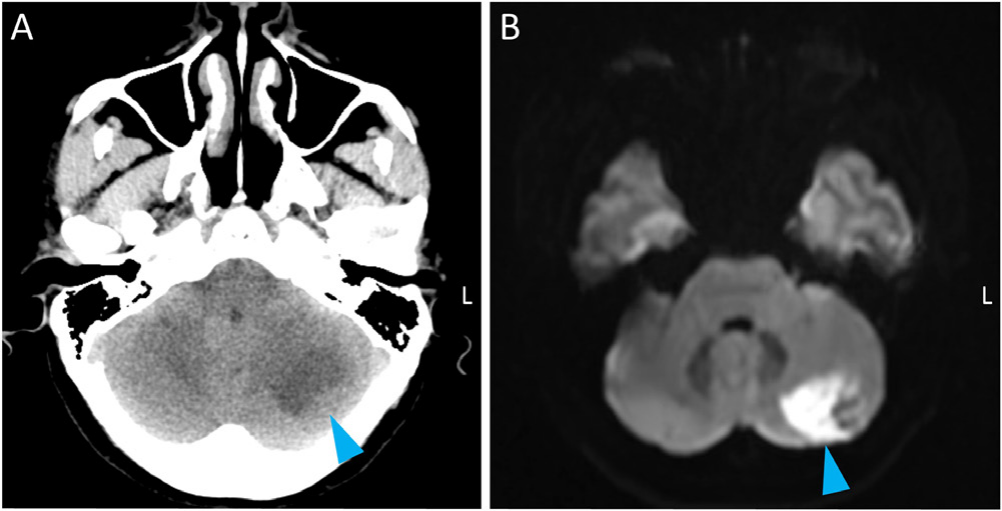
CT and MRI findings of acute left cerebellar infarction.

**Figure 2:**
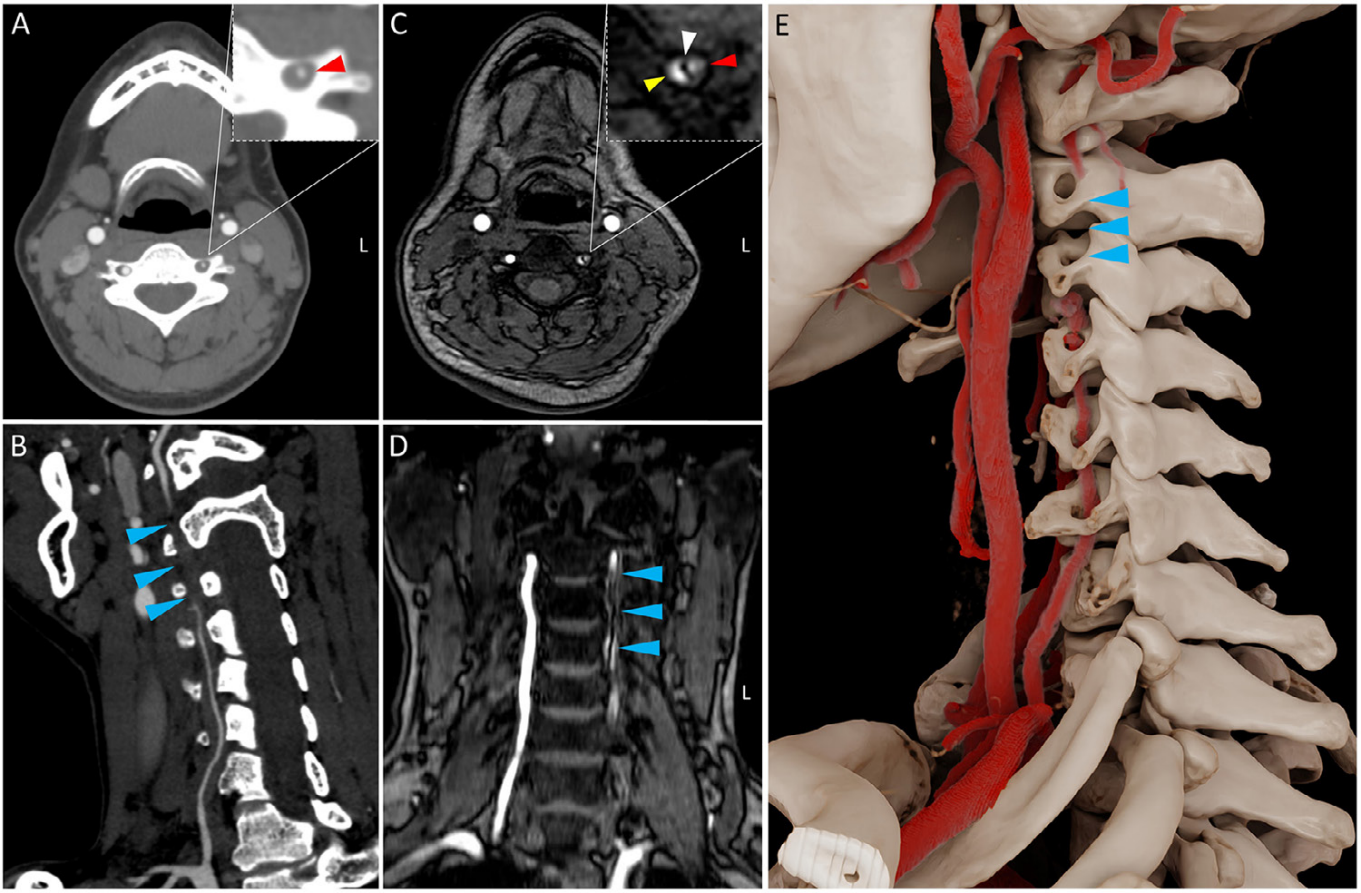
CTA and MRA findings of left vertebral artery dissection.

A 32‑year‑old woman presented with a persistent occipital headache that began the day after she experienced transient dizziness following a neck massage. The patient had no known risk factors for cerebrovascular disease. An unenhanced emergency head CT revealed hypodensity in the left cerebellum (Figure 1A, arrow). A brain MRI was performed, which showed hyperintensity on diffusion‑weighted imaging (DWI) in the left cerebellum, indicative of an acute infarction (Figure 1B, arrow). Magnetic resonance angiography (MRA) of the brain demonstrated no significant abnormalities in the major intracranial vessels. Subsequent cervical CT angiography (CTA) was performed for further vascular assessment, which demonstrated stenosis of the left vertebral artery at the V2 segment (Figure 2A) and occlusion at the V2–V3 junction, further evidenced by curved planar reformation and volume rendering (Figure 2B, 2E). Laboratory tests ruled out immune‑associated vasculitis. Ultimately, high‑resolution cervical MRA (time‑of‑flight) demonstrated the classic ‘double lumen sign’ (Figure 2C, red: true lumen; yellow: false lumen) with a displaced intimal flap (Figure 2C, white arrow), also observed in the coronal plane (Figure 2D, arrow), thus conclusively confirming the diagnosis of vertebral artery dissection (VAD).

## Comments

VAD is a significant cause of ischemic stroke in young adults, accounting for 8–11% of cases [1]. It often results from an intimal tear that leads to an intramural hematoma and can be triggered by minor trauma or occur spontaneously. The clinical presentation is frequently nonspecific, featuring headache, neck pain, and dizziness, which may result in diagnostic delays.

**Figure 3:**
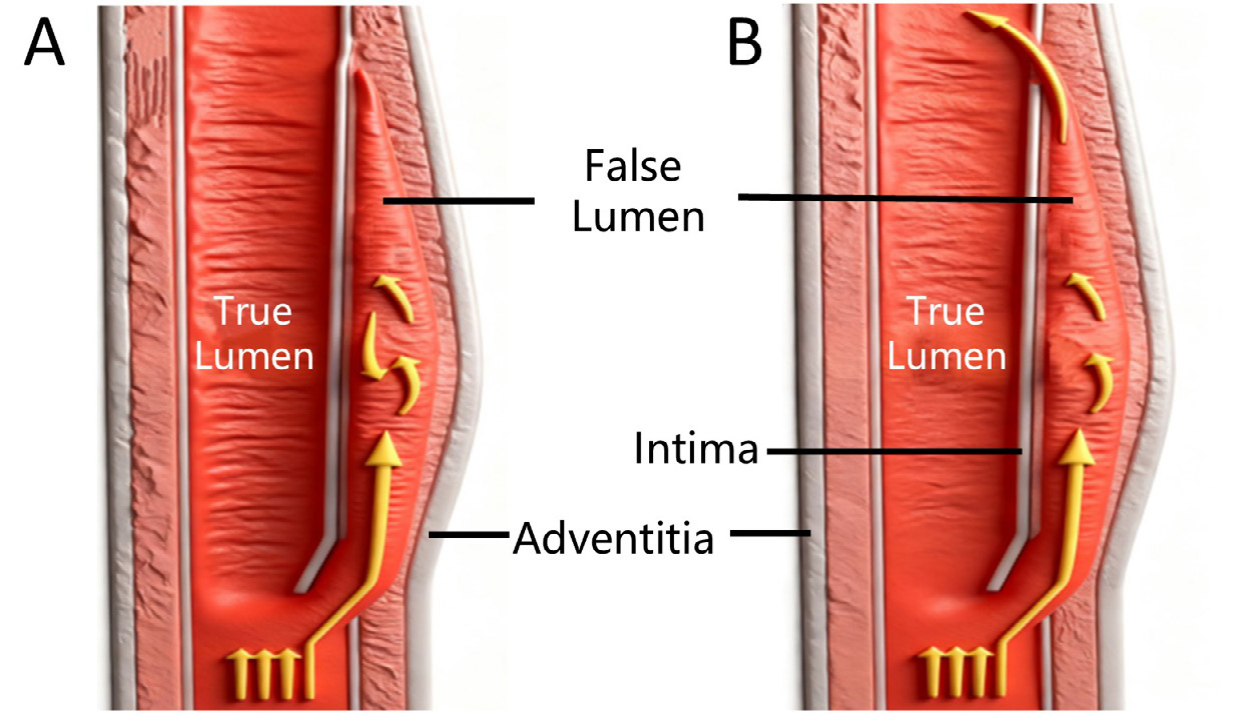
Diagnostic schematic illustrating arterial dissection findings.

A structured imaging pathway is critical for VAD diagnosis. Initial unenhanced brain CT excludes hemorrhage. Suspected posterior circulation infarction is confirmed by brain MRI with DWI, followed by intracranial MRA to rule out large vessel pathology. If unremarkable, evaluation shifts to the cervical vessels. Cervical CTA rapidly assesses patency but may only show stenosis/occlusion if the false lumen is thrombosed (Figure 3A), thereby missing the ‘double lumen sign’. In such cases, high‑resolution cervical MRA is crucial, as it delineates the intramural hematoma independent of false lumen flow (Figure 3B).

Accurate diagnosis of VAD is crucial, as thrombolysis is contraindicated due to the risk of hematoma expansion or hemorrhage. Anticoagulation is the preferred treatment, as evidenced by the successful management of this case with Rivaroxaban, Aspirin, and blood pressure control. This highlights the necessity for systematic imaging; when CTA is inconclusive, cervical MRA must be employed to confirm VAD, thereby guiding appropriate anticoagulation and preventing the dangers associated with thrombolysis.

In conclusion, maintaining a high clinical suspicion for vascular injury resulting from cervical mechanical stress, along with a systematic diagnostic approach, is crucial. If CTA yields inconclusive results, high‑resolution cervical MRA becomes essential for a definitive diagnosis. This imaging process contributes to appropriate anticoagulation therapy and to avoid potentially dangerous thrombolysis.
